# Association between Oestrogens Receptor Expressions in Breast Cancer and Comorbidities: A Cross-Sectional, Population-Based Study

**DOI:** 10.1371/journal.pone.0098127

**Published:** 2014-05-21

**Authors:** Laure de Decker, Mario Campone, Frederique Retornaz, Gilles Berrut, Anastasia Kabeshova, Florence Molinié, Olivier Beauchet

**Affiliations:** 1 Department of Therapeutic, EA 1156-12, Division of Geriatric Medicine, Nantes University Hospital, Nantes, France; 2 Department of Neuroscience, UPRES EA 4638, UNAM, Division of Geriatric Medicine, Angers University Hospital, Angers, France; 3 Ouest Cancer Institut, UMR-INSERM U892, Nantes, France; 4 Department of Departmental Geriatrics, Marseille, France; 5 Ouest Cancer Registry, Nantes, France; University of Texas Health Science Center at San Antonio, United States of America

## Abstract

**Background:**

Breast cancer with oestrogen receptor expression is common in older women. Several factors, such as age and reproductive hormone exposure, have been associated with oestrogen receptor expression in breast cancer. However, the association between comorbidities and the oestrogen receptor expression has been poorly studied. We hypothesized that there was an association between burden comorbidity and breast cancer with oestrogen receptor expression in older women.

**Objective:**

To determine whether oestrogen receptor expression in breast cancer was associated with burden comorbidity in community-dwelling women.

**Methods:**

A total of 1,707 women with breast cancer registered on the list of a breast cancer registry were included. The recorded data included: age, Charlson Comorbidity Index score≥1, breast cancer characteristics (coded according to the International Classification of Diseases for Oncology), and breast cancer pathological stage (the pathological-tumour-node-metastasis, Scarff Bloom Richardson, and hormonal status of oestrogen receptor, progesterone receptor, and human epidermal growth factor receptor).

**Results:**

Breast cancer with oestrogen receptor expression was identified in 1,378 patients (80·7%). The fully-adjusted logistic regression showed that oestrogen receptor expression was associated with Charlson Comorbidity Index score≥1 (odds ratio [OR] = 1·91,95%confidence interval [CI] = [1.01–3.61], P = 0·048), progesterone receptor expression (OR = 16·64, 95%CI = [11.62–23.81], P<0·001), human epidermal growth factor receptor (OR = 0·54, 95%CI = [0.34–0.84], P = 0·007), age (OR = 1.02, 95%CI = [1.00–1.03], P = 0.008), Scarff Bloom Richardson grade II and grade III (OR = 0·21with 95%CI = [0.10–0.44] and OR = 0·06 with 95%CI = [0.03–0.12], P<0·001).

**Conclusion:**

Our findings provide new data showing an independent positive association between burden comorbidity and breast cancer with oestrogen receptor expression. This result confirms that evaluation of oestrogen receptor expression in breast cancer should not be limited to hormonal factors stratified by age.

## Introduction

Breast cancer (BC) is the most commonly diagnosed cancer in women in the United States (US) and Europe [1]. The incidence rate for BC increases with age, explaining that 60% of diagnosis of BC occurred after 65 years and older [2]. Clinical, pathologic, and molecular features of BC are heterogeneous, thus BC has been classified into different subtypes [3]. One of these subtypes refers to immunohistochemical expression of hormonal receptors including oestrogens, progesterone, and the human epidermal growth factor receptor (HER) [4]. Hormonal receptor identification is important for the management of BC. For instance, BC with oestrogen receptor (ER) expression (ER+) may be treated with specific endocrine therapy and it has a better prognosis than BC without ER [5]. Thus, understanding which factors influence the expression of ER could be helpful for improving BC management.

Several factors, such as age and exposure to reproductive hormones, have been associated with ER+ in BC [3]. Compared to these factors, few studies have examined the association with comorbidities; that is, one or more other chronic or acute diseases in an individual with an index-disease (BC in our study) [4], [6]. Similar to the expression of ER and age that influence BC prognosis [6], comorbidities affect cancer mortality [7], [8]. For instance, comorbidities clearly impact the risk of death [9], [10], [11] and the risk of cancer recurrence [12]. Because of the age-related increase in BC with ER+ and chronic diseases [13], [14] it could be assumed that there is an association between comorbidity burden and ER+ in BC in older women.

Only a few studies have examined the association between comorbidities and BC with ER+. These studies reported mixed results [4], [15], [16]. Some studies failed to find an association [15], [16], whereas others reported significant associations [4], [16]. One explanation of the apparent discrepancy may be related to the definition and quantification of comorbidities in the same individual. Different tools have been specifically developed to score the comorbidities burden, or the number of chronic diseases and their severity [17]. The Charlson Comorbidity Index (CCI) is a standardized and validated method for scoring comorbidities and predicting mortality by weighting chronic conditions [18]. We hypothesized that there was an association between the comorbidities burden quantified using the CCI score and ER+ in BC in older women. The aim of this study was to determine whether BC with ER+ was associated with the CCI score in community-dwelling women.

## Materials and Methods

### Standard Protocol Approvals, Registrations, and Patient Consents

The study was conducted in accordance with the ethical standards set forth in the Helsinki Declaration (1983). The Angers Ethical Committee approved the study protocol and the study is in compliance with the Strengthening the Reporting of Observational Studies in Epidemiology statement guidelines. The institutional review board waived the need for written informed consent from the participants. Waiving of consent was authorized for this study according to French law.

### Participants

A total of 1,855 women with BC were included in this cross-sectional study. All participants were registered on three cancer registries involving the following French administrative areas: Doubs, Loire-Atlantique, and Tarn. The inclusion criterion for the study was BC diagnosis in 2007. The exclusion criterion was male gender. Information collected by the BC registry came from several databases involving public and private pathology laboratories and hospitals, the regional French National Health System, and Comprehensive Cancer Centres. Because analyses in the present study focused on ER status, we excluded 148 women (8.0%) who had unknown ER status. Therefore, full data were available for 1,707 (92·0%) women in the registry.

### Clinical Assessment

Data collected by the cancer registries and used in this study were age, comorbidities (the number of chronic diseases and their severity), and the BC characteristics coded according to the International Classification of Diseases for Oncology, 3^rd^ Edition. In addition, the BC pathological-tumour-node-metastasis (PTNM) was assessed using the recommendations of the 5th edition of Union for International Cancer Control. The stages have been grouped into three groups: the first group combined stage 0 and I tumours less than 2 cm without lymph node involvement, the second group combined stage II and III tumours comprising between 2·1 cm and 5 cm, with or without lymphadenopathy, and the third group corresponded to stage IV metastatic tumours. Furthermore, the Scarff Bloom Richardson (SBR) grade, modified by Elston and Ellis, was also measured because this classification of BC evaluates the “aggressiveness” of the tumour by taking into account the speed of tumour development and its management [12]. The hormonal status of ER, PR, and the HER was also recorded.

Comorbidities were scored using CCI. The CCI was validated in 1987 by ME Charlson [18] and consists of 18 groups of diseases with a weighted number assigned to each disease category [1]–[3], [13]. The weighted numbers are summed to obtain an overall score. Because a CCI score ≥1 defined comorbid condition [13], [18], this value was set as the cut-off point to separate women into the two groups for this analysis.

### Statistics

The participants' baseline characteristics were summarized using means and standard deviations or frequencies and percentages, as appropriate. Normality of the data distribution was evaluated using the Skewness-Kurtosis test. As the number of observations was >40 for each group, no transformations were applied to the variables of interest. Participants were separated into two groups based on the presence or absence of ER. Between-group comparisons were performed using an independent sample *t*-test or chi-square (χ^2^) test, as appropriate. Univariate and multiple logistic regressions were performed to examine the association between the presence of ER (dependent variable) and the CCI score≥1 (independent variable) adjusted for the participants' baseline characteristics. P-values <0.05 were considered statistically significant. All statistics were performed using SPSS (version 15·0; SPSS, Inc., Chicago, IL).

## Results

As shown in Table 1, a total of 1,378 (80·7%) patients had BC with ER+. These women were older (P<0·001) than those without ER expression. PTNM stages 0-I (P = 0·045) and IV (P = 0·014) were more prevalent only among participants with ER+ tumours. In addition, the expression of ER was associated with the presence of PR (P<0·001). The expression of HER and SBR grade were lower (P<0·001) in women with BC with ER+ compared to those without. There were no significant differences between the groups for the other characteristics.

**Table 1:** Comparison of baseline characteristics among breast cancer cases and oestrogen receptor status (n = 1,707).

	Total Population (n = 1,707)	Oestrogen receptor status		P-Value*
		Yes (n = 1,378)	No (n = 329)	
Age (years), mean±SD	60·8± 13.7	61·4±13.5	58·4±14.4	<**0·001**
Pathological-tumour-node-metastasis stage, n (%)				
0-I	895(54·1)	740(55·3)	155(48·9)	**0·045**
II-III	697(42·1)	555(41·5)	142(44·8)	0·283
IV	63(3·8)	43(3·2)	20(6·3)	**0·014**
Scarff Bloom Richardson grade, n (%)				
I	341(21·8)	331(26·1)	10(3·4)	<**0·001**
II	853(54·6)	735(57·9)	118(40·5)	<**0·001**
III	367(23·5)	204(16·1)	163(49·5)	<**0·001**
Progesterone receptors, n (%)	1188(69·6)	1128(81·9)	60(18·2)	<**0·001**
Human epidermal growth factor receptor, n (%)	183(10·7)	101(8·0)	82(26·9)	<**0·001**
Charlson Comorbidity Index, ≥1, n (%)	153(9.0)	129(9.4)	24(7.3)	0·283

* Based on independent samples *t*-test or chi-square test with *P* significant <0·05.

Significant P-values (P<0·05) are indicated in bold.

Table 2 displays the results of the univariate and multiple logistic regression models. The univariate model showed that age (odds ratio [OR] = 1·02, P<0·001), PTNM stage 0-I (OR = 1·29, P = 0·04), PTNM stage IV (OR = 0·49, P = 0·011), SBR grade I (OR = 9·90, P<0·001), SBR grade II (OR = 2·01, P<0·001), SBR grade III (OR = 0·15, P<0·001), PR+ (OR = 20·31, P<0·001), and HER+ (OR = 0·24, P<0·001) were significantly associated with ER+ tumours. No significant association was observed between ER+ and CCI score≥1 (OR = 1·31, P = 0·24). The fully adjusted logistic regression showed that ER+ tumours were inversely associated with SBR grade II (OR = 0·21, P<0·001), grade III (OR = 0·06, P<0·001) and HER+ (OR = 0·54, P = 0·007), and positively associated with age (OR = 1·02, P = 0·008), PR+ (OR = 16·64, P<0·001), and CCI score≥1 (OR = 1·91, P<0·048).

**Table 2:** Univariate and multiple logistic regression models showing the association between oestrogens receptor expression (dependent variable) and Charlson Comorbidity Index (independent variable) adjusted for clinical characteristics (n = 1,707).

	Unadjusted model			Fully adjusted model		
	OR	95% CI	P-value*	OR	95% CI	P-value*
Age	**1·02**	[1·01–1·02]	**<0·001**	**1.02**	[1·00–1·03]	**0.008**
Pathological-tumour-node-metastasis stage						
0-I	**1·29**	[1·01–1·65]	**0·04**	(Ref†)	-	-
II-III	0·87	[0·68–1·12]	0·28	0·85	[0·60–1·22]	0.85
IV	**0·49**	[0·29–0·85]	**0·011**	0·65	[0·29–1·44]	0.65
Scarff Bloom Richardson grade						
I	**9·90**	[5·21–18·84]	**<0·001**	(Ref†)	-	-
II	**2·01**	[1·55–2·61]	**<0.001**	**0·21**	[0.10–0.44]	**<0·001**
III	**0·15**	[0·11–0·20]	**<0.001**	**0·06**	[0.03–0.12]	**<0·001**
Progesterone receptors	**20·31**	[14·87; 27·73]	**<0·001**	**16·64**	[11·62–23.81]	**<0·001**
Human epidermal growth factor receptor	**0·24**	[0.17–0.33]	**<0·001**	**0·54**	[0·34–0·84]	**0·007**
Charlson Comorbidity Index, ≥1	1·31	[0.83–2.06]	0.240	**1·91**	[1·01–3·61]	**0.048**

OR: odds ratio; CI: confidence interval.

* Between-group comparison based on simple *t*-test or Chi-square test.

† : Score = 1 used as reference level.

Significant P-values and OR (P<0·05) are indicated in bold.

## Discussion

The main finding of our study is that a CCI score≥1 is associated with the expression of ER in women with BC. This association was independent of age, other hormonal receptors, PTNM stages, and SBR grades. Furthermore, the grade of tumors and the presence of HER were significantly associated with ER-, whereas the expression of PR and age were related to ER+. Finally, our findings indicate that PTNM stages were not associated with ER expression.

The comorbidity burden quantified by the CCI score was an independent factor associated with the expression of ER in the sample of studied women. To the best of our knowledge, no previous studies have examined the association between the comorbidity burden and ER+ in BC. It has been reported that the comorbidity burden is associated with increased mortality and affects prognosis and treatment in all hormonal types of BC [19], [20], [21]. Most previous contradictory studies likely focused on specific cormorbidities such as the metabolic syndrome (defined as an association of high blood pressure, obesity, dyslipidaemia, and diabetes) or obesity alone for two main reasons [4], [15], [16]. Firstly, obesity and metabolic syndrome are associated with hormonal status, and in particular, with the presence of oestrogen, which is a reproductive hormone that has a determinant role in BC aetiology [3]. Secondly, cardiovascular comorbidities (including metabolic syndrome and obesity) are the most prevalent diseases in women with BC [22]. Unlike these previous studies, we chose to examine the association with the CCI score because it integrates the number and the severity of chronic disease, and because it is associated with the risk of death [7]. The CCI score is a better marker of an individual's morbidity status because it reflects the global comorbidities burden, rather than a single aspect of the adverse effect of one comorbidity. Thus, we suggest that the CCI score, which includes the effects of all comorbidities and not only those of hormonal comorbidities, appears to be a consistent measure of the association between comorbidities and ER+ in BC. The pathophysiological support of the association between comorbidities and ER+ reported in our study is not yet fully elucidated. It could be related to the fact that ER belongs to the super-family of the nuclear hormone receptors involved both in cancers and other morbidities [23]. For instance, the ER+ has been shown in comorbidities such as osteoporosis, Alzheimer's disease, or cardiovascular diseases and breast and endometrial cancers [23]. Furthermore, it has been reported that there is a polymorphism in steroid hormone genes in the most frequent morbidities including cardiovascular and kidney diseases, and cancers [24]. Aromatase gene polymorphisms have also been related to comorbidities such as osteoporosis, Alzheimer's disease, or cardiovascular diseases and several cancers [24].

Age was significantly associated with ER+ in our study. This association may be considered as clinically relevant. Indeed, the OR was calculated at 1.02, which corresponds to an increase of 2% per year of the association between age and ER+ for a woman of 60.8 years old. Considering periods of 10 years, it means that 20% of women aged 71 years old, or 40% of women aged 81 years old with BC have ER+. This result is in concordance with previous studies in which the number of BC with ER expression increased with age [5], [10], [13], [14]. However, compared to the present study, comorbidities were not used as an independent variable in these previous studies. Aging is a heterogeneous continuous process [25] characterized by the accumulation of comorbidities, explaining a strong relationship between these two variables. We examined this relationship in our population and found, as previously reported, that the CCI score increased significantly with age (data not shown) [6], [8]. However, our results underscored that, despite the close relationship between age and comorbidity, there was a specific and independent association of comorbidity burden with ER+.

Regarding the other hormonal status in our study, BC with PR expression was positively associated with ER+. This result is in concordance with previous studies [5], [26]. This positive association is the most frequent phenotype and is related to a higher concentration of endogenous steroids [27], [28]. In our study, the absence of HER expression and histological lower grading of BC also positively associated with ER+, which is in agreement with previous published data [29], [30], [31] and confirms that the population studied is a representative population of BC patients.

Some limitations of the study should to be considered. First, the cross-sectional design of the present study may limit exploring the association between BC with ER+ and comorbidities compared to a prospective cohort design and thus prevent causal inferences. Second, although we were able to control for several characteristics that may have modified the association, residual potential confounders might still be present. Indeed, we were not able to control for factors related to hormonal status, such as nulliparity, metabolic syndrome, age at first birth, and menarche. However, studies showed that a systemic exposure of these factors was associated with the hormonal status of BC [3].

In conclusion, our study provides new data showing an independent positive association between a CCI score≥1 and BC with ER+. This result confirms that evaluation of ER expression in BC should not be limited to hormonal factors stratified by age [3]. The comorbidity burden as a determinant risk factor of hormonal status in BC should open further areas of research.
